# Expression of Programmed Cell Death-Ligand 1 (PD-L1) in Astrocytic Tumors and Its Correlation With Histopathological Grade and Proliferative Index (Ki-67): A Cross-Sectional Study

**DOI:** 10.7759/cureus.79728

**Published:** 2025-02-26

**Authors:** Namita Singh, Ranjana Giri, Prita Pradhan, Diptiranjan Satapathy, Ipsita Debata

**Affiliations:** 1 Pathology, Kalinga Institute of Medical Sciences, Bhubaneswar, IND; 2 Neurosurgery, Kalinga Institute of Medical Sciences, Bhubaneswar, IND; 3 Community and Family Medicine, Kalinga Institute of Medical Sciences, Bhubaneswar, IND

**Keywords:** astrocytic tumors, grade, ki-67, pd-l1, programmed cell death-ligand 1, survival

## Abstract

Background

Multimodality therapies for gliomas are associated with poor outcomes. Tumor cells display immune evasion by activating immune checkpoint molecules such as programmed cell death-ligand 1 (PD-L1). It could be linked with the grade proliferative index (Ki-67) serving as a potential target for immunotherapy. This study aimed to investigate the expression of PD-L1 in astrocytic tumors and correlate it with histopathological grade and Ki-67.

Methodology

This cross-sectional analytical study was conducted in the Department of Pathology of a tertiary medical college for four years between September 2018 and August 2022. A total of 43 cases of biopsy-proven glial tumors were included in the study after obtaining informed consent from all participants whose biopsies were considered. Clinicoradiological details were documented. Hematoxylin and eosin (H&E) slides were reviewed for histopathological grade and morphology. PD-L1 expression and Ki-67 labeling Index were studied immunohistochemically, followed by the correlation of PD-L1 with clinicopathological parameters, Ki-67 LI, and patient survival was also noted. Data were compiled into Microsoft Excel (Microsoft Corp., Redmond, WA, USA) and analyzed using SPSS Statistics for Windows, Version 21.0 (IBM Corp., Armonk, NY, USA). Descriptive data were interpreted as mean, frequencies, and percentages. Spearman’s rank correlation test was used to study the correlation between PD-L1 and Ki-67. Association was tested using the chi-square test. The impact of PD-L1 on the survival of patients was also investigated by Kaplan-Meier survival analysis.

Results

A total of 43 patients were included with 31 males and 12 females (mean age = 46.14 years). PD-L1 expression and Ki-67 LI showed a significant relationship with advanced age (p = 0.010), histological grade (p = <0.001), presence of cellular atypia (p < 0.001), necrosis (p = <0.001), and microvascular proliferation (p < 0.001). PD-L1 showed a positive correlation with Ki-67 (r_s _= 0.390; p = 0.009) and shorter survival (p = 0.006).

Conclusions

Greater PD-L1 expression in astrocytic tumors is associated with higher grade and Ki-67 LI. Poor survival with PD-L1 positivity is likely reflective of the increased aggressiveness conferred by the PD-L1-induced immune evasion. PD-L1, a negative predictive biomarker, may serve as a novel target in immunotherapy for gliomas.

## Introduction

Central nervous system (CNS) tumors are the 10th most common tumors worldwide, accounting for 2.4% of all malignancies [1]. Gliomas are the most common type of primary brain tumor of CNS [2]. Though multimodal therapies such as surgery, radiotherapy, and chemotherapy have been suggested, the overall survival of glioma patients remains dismal. The five-year survival rate is very low in patients with glioblastoma [3]. Unlike other tumors, molecular targeted therapies for high-grade gliomas produced very limited advances in the life expectancies of patients. This is attributable to poor penetration of the blood-brain barrier by therapeutic agents and rapidly developing drug resistance [4,5]. Therefore, it is necessary to explore new therapeutic approaches for improvement in glioma patients.

Glioma creates an immunosuppressive environment and evades immunosurveillance. Programmed cell death 1(PD-1)/programmed cell death-ligand 1 (PD-L1) is a classic immune checkpoint [6]. PD-L1 is expressed by tumor cells which bind with PD-1 on T cells, B cells, dendritic cells, and natural killer (NK) cells and enable escape of the tumor cells from immune attack via immunosuppression. Tumor immunity can be promoted by blocking the binding between PD-1 and PD-L1, which can serve as an emerging immunotherapy modality. Blocking of the PD-L1/PD-1 pathway can prove to be a novel and promising immunotherapeutic strategy for glial tumors [2]. Histopathological grade and proliferative index (Ki-67) are the potential biological prognostic markers of gliomas. Thus, this study aimed to investigate the immunohistochemical (IHC) expression profile of PD-L1 on astrocytic tumors and its correlation with histopathological grade and proliferative index (Ki-67). In addition, the impacts of PD-L1 on survival were investigated.

## Materials and methods

Study design, setting, and population

The cross-sectional analytical study was conducted in the Department of Pathology of a tertiary medical college for four years between September 2018 and August 2022. A total of 43 cases of biopsy-proven glial tumors were included in the study after informed consent was obtained from all participants whose biopsies were considered for inclusion.

Inclusion criteria

All biopsy-proven glial tumors diagnosed during the study period were included. Formalin-fixed paraffin-embedded blocks of histopathologically proven cases of glial tumors were included, both prospectively and retrospectively from the archives.

Exclusion criteria

Cases of gliosarcoma, mixed glioneuronal tumors, technically suboptimal tissue, recurrent cases, and those with prior chemo/radiation therapy were excluded from the study.

Sample collection

Consecutive hematoxylin and eosin sections were stained and slides were reviewed for histomorphological features, including histological type and grade of tumors; the degree of cellularity, atypia, and mitosis; presence or absence of microvascular proliferation; and necrosis. The corresponding sections from the paraffin block with viable and highly cellular tumor areas (including necrosis if present) were chosen for IHC.

Immunohistochemical analysis

IHC for PD-L1 and Ki-67 was performed on deparaffinized sections taken on albumin-coated slides using two-step indirect methods. Pre-diluted ready-to-use monoclonal antibodies of PD-L1, P-P001-30 (Clone QR001, Berlin, Germany) and Ki-67, CAT-P-K001-30 (Clone QR015, Berlin, Germany) were used. The placenta and tonsil were taken as a positive control for the former and latter, respectively. For negative control, the same sections were used omitting the primary-antibody step. The observations were taken independently by two pathologists blinded to the clinical data. The discrepant cases were reviewed and evaluated over a multiheader microscope with an expert pathologist for final consensus.

Evaluation of PD-L1

In the present study, PD-L1 staining with membranous and/or cytoplasmic/fibrillary pattern in >1% of cells was considered positive PD-L1 expression [7]. The location, area (%), pattern of staining (membranous vs. cytoplasmic/fibrillary), and intensity (1+, 2+, and 3+) of staining were also documented.

Evaluation of Ki-67

A hotspot (area with the highest density of immunostained nuclei) was selected and adjacent fields were counted to include 1,000 nuclei. Distinct nuclear staining of tumor cells was recorded as positive. Ki-67 labeling index (LI) was recorded as a percentage of positively stained nuclei in 1,000 tumor cells.

Data analysis

Data were compiled into Microsoft Excel (Microsoft Corp., Redmond, WA, USA) and analyzed using SPSS Statistics for Windows, Version 21.0. (IBM Corp., Armonk, NY, USA). Descriptive data were interpreted as mean, frequencies, and percentages. Spearman’s rank correlation test was used to study the correlation between PD-L1 and Ki-67. Association was tested using the chi-square test. The impact of PD-L1 on the survival of patients was also investigated using Kaplan-Meier survival analysis. A p-value ≤0.05 was considered statistically significant.

Ethical considerations

Institutional ethical clearance (approval number: KIIT/KIMS/IEC/493/2020) was obtained before study initiation. The study was conducted according to the Declaration of Helsinki. All participants whose biopsies were considered for the study provided informed consent. After informed consent was obtained, detailed clinical and radiological information was documented.

## Results

A total of 43 patients were found to satisfy the inclusion criteria with histopathologically proven astrocytic tumors. There were 31 (72.09%) males and 12 (27.91%) females. The age range was 3-73 years. The frontal lobe was the most frequent site (48.8%, 21) followed by the parietal lobe (20.9 %, 9) while one-third of cases had more than one lobe involvement. Right-sided lesions were most frequent (48.8%, 21) while 4.6% (2) of cases showed bilateral involvement. The study included 15 (34.8 %) cases of low-grade glioma (WHO grade I and II) and 28 (65.2 %) high-grade gliomas (WHO grade III and IV).

PD-L1 and Ki-67 expression in glioma

**Figure 1:**
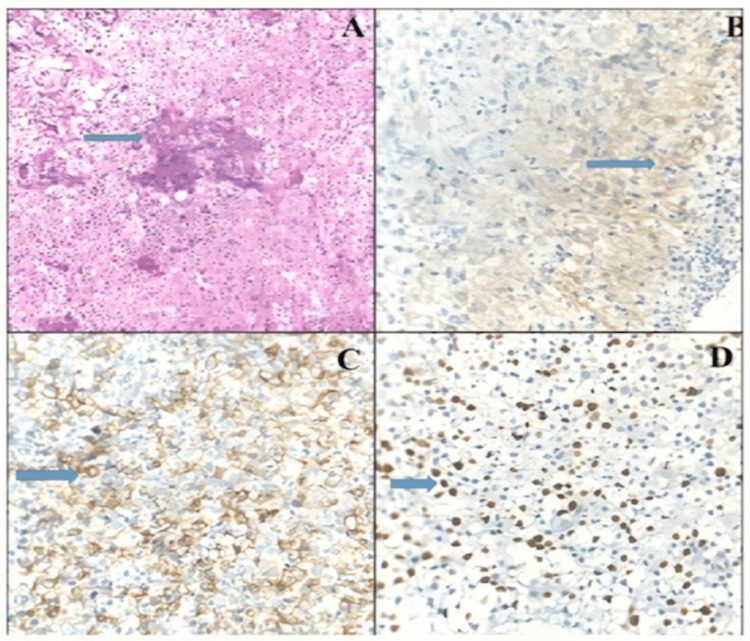
(A) A case of glioblastoma (IV) showing highly cellular tumor with atypia and microvascular proliferation (blue arrow) (H&E 400×). (B) PD-L1 immunostaining showing 1+ to 2+ intensity with fibrillary pattern in a case of glioma (blue arrow) (PD-L1 400×). (C) Membranous pattern (3+) (blue arrow) in a case of anaplastic astrocytoma (III) (PD-L1 400×). (D) High Ki-67 labeling index (blue arrow) in a case of glioblastoma (IV) (Ki-67 400×). PD-L1: programmed cell death-ligand 1; H&E: hematoxylin and eosin

PD-L1 was found to be positive in 58.14% (25/43) cases with percentage expression ranging from 1% to 90% (mean = 11.14 ± 20.77%). The intensity was mostly 2+ to 3+ in most cases (15/25). The most common staining pattern was cytoplasmic/fibrillary which was seen in 80% of cases (20/25). The Ki-67 LI was found to be positive in 95.3% of cases ranging from 0% to 80% (mean = 22.65 ± 24.28%). Both PD-L1 and Ki-67 were much higher in tumor tissue than adjacent normal brain parenchyma (Figure 1). The highest staining was seen in the high cellularity regions with maximum expression near blood vessels and near areas of necrosis. Stronger staining intensity was more frequently seen with higher-grade tumors. An occasional case showed PD-L1 expression even in tumor-infiltrating lymphocytes (TILs) and tumor cells.

PD-L1 expression and Ki-67 LI showed a statistically significant relationship with age, histological grade, presence of cellular atypia, necrosis, and microvascular proliferation but not with sex, site, size, or laterality (Table 1). Greater PD-L1 expression and higher Ki-67 LI were associated with the higher age of the patient, higher histologic grade, and greater degree of cellular atypia. It was also significantly associated with the presence of necrosis and microvascular proliferation.

**Table 1:** Correlation of PD-L1 and Ki-67 with clinicopathological parameters. *: Two cases were bilateral and were excluded from analysis. Association was tested by the chi-square test. P-value <0.05 was considered statistically significant. PD-L1: programmed cell death-ligand 1; MVP: microvascular proliferation

Parameters	Category	PD-L1 expression (%)		P-value	Ki-67 expression (%)		P-value
		Negative	Positive		Low	High	
Age (years)	<46 (n = 19)	11	8	0.050	10	9	0.010
	>46 (n = 24)	7	17		4	20	
Gender	Female (n = 31)	15	16	0.160	11	20	0.510
	Male (n = 12)	3	9		3	9	
Lobes involved	Single (n = 18)	9	9	0.360	7	11	0.510
	Multiple (n = 25)	9	16		7	18	
Size (cm)	<3 (n = 29)	12	17	0.930	9	20	0.750
	>3 (n = 14)	6	8		5	9	
Laterality^*^	Left (n = 20)	8	12	0.850	8	12	0.270
	Right (n = 21)	9	12		5	16	
Histologic grade (WHO)	I/II (n = 15)	10	5	0.020	12	03	<0.001
	III/IV (n = 28)	8	20		2	26	
Necrosis	Absent (n = 19)	12	7	0.010	13	6	<0.001
	Present (n = 24)	6	18		1	23	
MVP	Absent (n = 19)	12	7	0.010	13	6	<0.001
	Present (n = 24)	6	18		1	23	
Cellularity	Low (n = 27)	13	14	0.280	13	17	0.220
	High (n = 16)	5	11		4	12	
Atypia	Absent (n = 12)	9	3	0.010	9	3	<0.001
	Moderate/Marked (n = 31)	9	22		5	26	

Relationship between PD-L1 and Ki-67 LI

**Figure 2:**
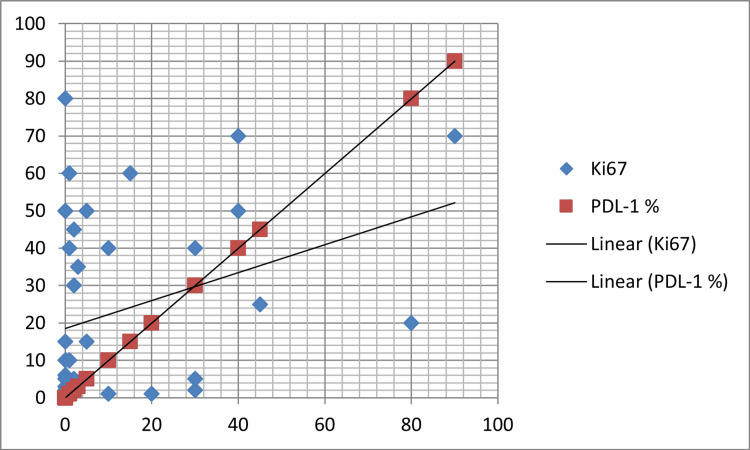
Scatter diagram showing a significant relationship between PD-L1 and Ki-67. PD-L1: programmed cell death-ligand 1

On Spearman’s correlation, PD-L1 showed a positive correlation with Ki-67 LI (r_s_ = 0.390; p = 0.009) implying greater PD-L1 expression was significantly associated with higher Ki-67 LI (Figure 2, Table 2).

**Table 2:** Relationship between PD-L1 and Ki-67 LI. Association was tested by chi-square test. P-value <0.05 was considered statistically significant. PD-L1: programmed cell death-ligand 1; LI: labeling index

Ki-67	PD-L1 –ve (n = 18)	PD-L1 +ve (n = 25)	P-value
<4% (n = 14)	9	5	0.040
>4% (n = 29)	9	20	

Survival analysis of PD-L1-positive vs. PD-L1-negative cases

**Figure 3:**
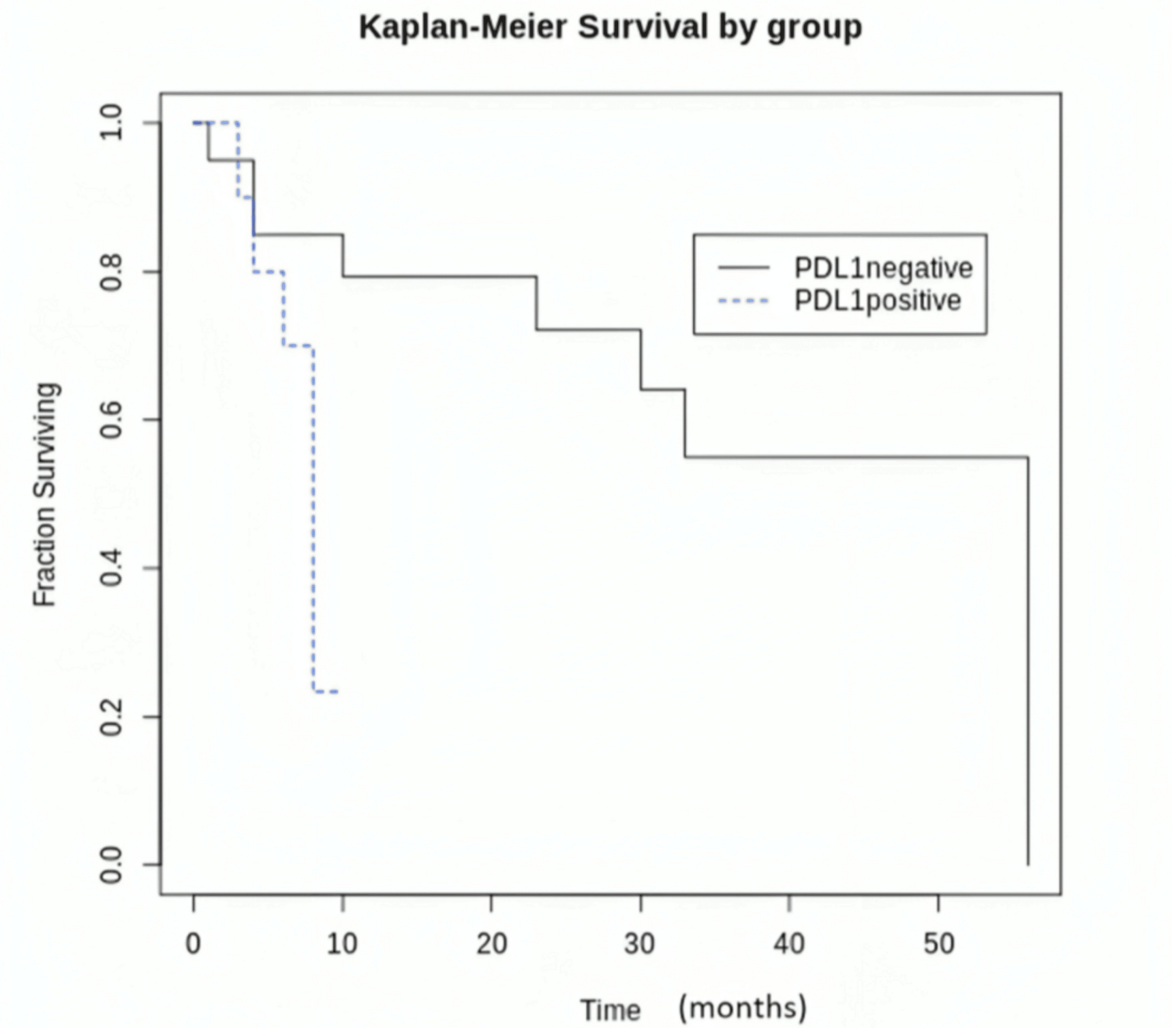
Kaplan-Meier survival analysis comparing the survival between PD-L1-positive and PD-L1-negative glioma cases. PD-L1: programmed cell death-ligand 1

On the log-rank test, PD-L1-positive gliomas showed a significantly shorter survival compared to PD-L1-negative cases (p = 0.006). The Kaplan-Meier Survival curve is depicted in Figure 3.

## Discussion

Glioma cells have a remarkable ability to evade immune attacks by creating an immunosuppressive environment. PD-1PD-L1, which serves as an immune classic immune checkpoint, helps tumor cells escape immune surveillance [2,6]. This study aimed to evaluate PD-L1 expression in gliomas and its relationship with grade and Ki-67 LI. The study proved the hypothesis that PD-L1 shows higher expression in cases of high-grade gliomas. Greater PD-L1 expression associated with higher age, higher WHO grade (III, IV), and greater degree of atypia was further highlighted by its association with necrosis and microvascular proliferation. The latter features are more frequently associated with the more aggressive behaving higher grade of gliomas. An occasional case was also associated with the expression of PD-L1 on TILs. PD-L1 expression in about 60% of cases, akin to the present study was found to be in diverse studies [2,8,9]. Higher levels of PD-L1 in high-grade gliomas, on the one hand, reflect the worse forms of tumor and, on the other hand, they offer a novel target for PD-L1 blockade.

This study showed the moderate intensity of staining with cytoplasmic pattern being most frequent with increased expression around blood vessels and necrosis. Similar findings have been documented in the literature by Zeng et al., Wilmotte et al., and Shukla et al. [6,8,9]. Xue et al. highlighted that PD-L1 in gliomas is significantly associated with the expression of vascular endothelial growth factor as well as tumor grade and Ki-67 LI. The perivascular PD-L1 expression could be attributed to the angiogenic potential. Similar to the present study, other studies have found a stronger intensity of staining in higher-grade gliomas [2]. However various studies have documented lower intensities [10]. The reason for these variable staining intensities and patterns is likely because of the different clones of monoclonal primary antibodies used by the different studies, including SP263, CAL10, and 5H1, while in this study QR001 was used [2,6,9,10]. The correlation of PD-L1 with Ki-67 confirms the hypothesis that greater PD-L1 is significantly associated with a higher proliferation index. This observation along with the PD-L1 expression on TILs and its association with poor survival points to a likely possibility of the reason for higher aggressiveness in higher-grade gliomas as discussed by Samman et al. [11]. The PD-L1-induced suppression of immunity by action on tumor cells, T cells, and antigen-presenting cells highlights the loss of cytotoxicity in T cells due to inhibition of cell activation. It suppresses the inflammatory cytokine release [11-13].

Glioblastoma is a tumor with aggressive behavior and a dismal prognosis. High PD-L1 and Ki-67 are negative prognostic biomarkers and may benefit from immunotherapeutic interventions [11]. Ki-67 is seen in dividing cells and offers an indicator of cellular replication [14,15]. Studies have highlighted higher Ki-67 in the gliomas increases the severity of malignancy. It helps in grading as well as prognostication. One of the difficulties encountered in this study is the lack of a standard method for evaluating PD-L1 expression in gliomas (Table 3). The cut-offs range from 1% to 5% for positivity.

**Table 3:** Variation in PD-L1 evaluation in gliomas. PD-L1: programmed cell death-ligand 1

Authors	Year	Country	Sample size (n)	Cut-off for PD-L1	Pattern staining	Ki-67
Han et al. [10]	2016	Korea	54	>5%	Membranous and cytoplasmic	-
Samman et al. [11]	2021	Egypt	30	>1%	Diffuse fibrillary and membranous	-
Shukla et al. [9]	2021	India	30	>1%	Diffuse and heterogeneous	-
Zeng et al. [6]	2016	China	229	>5%	Cytoplasmic or membranous	-
Xue et al. [2]	2017	China	64	>5%	Cytoplasmic or membranous	>10%
Berghoff et al. [16]	2015	Austria	135	>5%	Diffuse fibrillary or membranous	-
Ndoum et al. [7]	2016	USA	94	>1%	Membranous	-
Pratt et al. [17]	2019	USA	125	>5%	Membranous	-
Lee et al. [18]	2017	Korea	115	>5%	Membranous and fibrillary	-

The present study holds merit in being one of the few studies worldwide correlating PD-L1 in glioma and correlating it to Ki-67 LI. There is limited data from the Indian population regarding PD-L1 expression [9]. This study has not just evaluated the percentage of PD-L1 expression but also the intensity and the expression patterns. Despite the limited sample size, the study highlights the morphological aspects as well as incorporates the survival data. Limitations of the study include the limited number of cases of each subtype precluding a detailed subgroup analysis. Being a manual evaluation there is scope for significant subjectivity. PD-L1 expression on TILs and their populations have not been studied in detail. Further taking 1% as a cut-off makes false negatives a possibility because PD-L1 expression can be a focal marker. Lastly, being a retrospective study, follow-up data are not available in all the cases.

## Conclusions

This study has highlighted the expression patterns of PD-L1, an immune checkpoint, in various gliomas and its relevance. Greater PD-L1 expression in astrocytic tumors, particularly those with higher grade, and their association with higher age, greater cytologic atypia, necrosis, microvascular proliferation, and high Ki-67 and poor survival is likely reflective of the increased aggressiveness conferred by the PD-L1-induced immune evasion. PD-L1, a negative predictive biomarker, may serve as a novel target in immunotherapy for glioma. Larger prospective studies with detailed follow-up and correlation with the TIL population characterization are essential for a better understanding of the disease.
